# Prenatal stress causes intrauterine inflammation and serotonergic dysfunction, and long-term behavioral deficits through microbe- and CCL2-dependent mechanisms

**DOI:** 10.1038/s41398-020-00876-5

**Published:** 2020-06-16

**Authors:** Helen J. Chen, Adrienne M. Antonson, Therese A. Rajasekera, Jenna M. Patterson, Michael T. Bailey, Tamar L. Gur

**Affiliations:** 1 Department of Psychiatry and Behavioral Health, The Ohio State University Wexner Medical Center, Columbus, OH USA; 2 Department of Neuroscience, The Ohio State University Wexner Medical Center, Columbus, OH USA; 3 Institute for Behavioral Medicine Research, The Ohio State University Wexner Medical Center, Columbus, OH USA; 4 Biosciences Division, College of Dentistry, The Ohio State University, Columbus, OH USA; 5 Environmental Health Sciences Division, College of Public Health, The Ohio State University, Columbus, OH USA; 6 Center for Microbial Pathogenesis, The Research Institute, Nationwide Children’s Hospital, Columbus, OH USA; 7 Department of Pediatrics, The Ohio State University Wexner Medical Center, Columbus, OH USA; 8 Obstetrics and Gynecology, The Ohio State University Wexner Medical Center, Columbus, OH USA

**Keywords:** Molecular neuroscience, Autism spectrum disorders

## Abstract

Prenatal stress (PNS) is associated with neuropsychiatric disorders in offspring, including anxiety, depression, and autism spectrum disorders. There is mounting evidence that these behavioral phenotypes have origins *in utero*. Maternal microbes, inflammation, and serotonergic dysfunction have been implicated as potential mediators of the behavioral consequences of PNS; whether and how these systems interact is unclear. Here, we examine the effects of PNS *in utero* using late-gestation maternal restraint stress in wild-type (WT), germ-free (GF), and CCL2^−^^/^^−^ genetic knock-out (KO) mice. In WT mice, PNS leads to placental and fetal brain inflammation, including an elevation in the chemokine CCL2. This inflammation is largely absent in GF mice, indicating the critical role of maternal microbes in mediating immune processes *in utero*. Furthermore, PNS in the absence of CCL2 failed to increase pro-inflammatory cytokine IL-6 in the fetal brain. PNS offspring also exhibited deficits in sociability and anxiety-like behavior that were absent in CCL2^−^^/−^ PNS offspring. Tryptophan and serotonin (5-HT) were elevated in the WT PNS placenta, but not in CCL2^−^^/−^ and GF animals. Altogether, these findings suggest that a complex interaction between maternal microbes, inflammation, and serotonin metabolism regulates the emergence of behavioral abnormalities following PNS.

## Introduction

Prenatal stress (PNS) has been linked with adverse neuropsychiatric outcomes^1,2^. Children of women who experience psychosocial stress during pregnancy are at elevated risk of depression, anxiety, and autism spectrum disorders^1–3^. This phenomenon is supported by findings from rodent models, in which PNS leads to anxiety-like behavior, social deficits, and depressive-like behavior in the offspring^4–8^. However, the mechanisms underlying possible fetal programming during PNS are still being elucidated.

Perturbation of the maternal gut microbiome has been implicated as a mechanism through which PNS can influence neurodevelopment^9–11^. There is mounting evidence that commensal microbiota intimately interact with and shape both the developing immune system and the brain^12,13^. Toll-like receptors (TLR) present on immune cells and trophoblast cells of the placenta^14,15^ allow for microbes or microbial components to signal across fetal tissue, initiating an inflammatory cascade and resulting in the production of pro-inflammatory cytokines and chemokines^15–17^. These interactions are necessary for the maturation of the immune system, as germ-free (GF) mice have diminished hematopoietic capacity and blunted cytokine production^18,19^. While PNS has been shown to upregulate expression of pro-inflammatory cytokines in the placenta and fetal brain^5,6,20^, the interplay between maternal microbes and inflammation in mediating the programming effects of PNS is unclear.

C-C motif chemokine ligand 2 (CCL2) is a chemokine that primarily functions in the recruitment of leukocytes to propagate an inflammatory response^21^ and recruits monocytes to the brain to elicit an anxiogenic response during stress^22,23^. CCL2 is also highly expressed in uterine and placental tissue^24–26^; whether CCL2 plays a role in fetal neurobiological and behavioral programming is unknown. Therefore, the contribution of placental and fetal brain CCL2 to neurodevelopmental and behavioral outcomes is an important area for investigation, especially in the context of PNS.

The potential for inflammatory signals to alter neurodevelopment is well documented, and can occur directly through the damaging effects of cytokine signaling^27^, or indirectly through various mechanisms. These include alterations to the hypothalamic–pituitary–adrenal axis, disruptions of offspring gut microbial colonization, or changes to offspring neuroimmune function (as Reviewed in ref. ^28^). Another possible mechanism is by modulating serotonergic synthesis, transport, and breakdown^29,30^. We have previously shown that exposure to restraint stress during pregnancy results in elevated plasma levels of serotonin (5-HT) in male offspring concurrent with sociability deficits and cortical inflammation^4^. Dysfunction of the immune and serotonergic systems is also observed in patients with autism spectrum disorders^31,32^. There is evidence to suggest that a dysfunctional serotonergic phenotype has intrauterine origins, as the placenta is the primary source of 5-HT for the fetal brain prior to the development of serotonergic neurons between embryonic days 12–14^33,34^. Directly stimulating a maternal immune response during pregnancy is sufficient to alter placental 5-HT synthesis^35^. Indirect perturbations, including PNS, can result in altered fetal serotonin synthesis as evidenced by elevated concentrations of 5-HT in the fetal brain^36^. Furthermore, administration of bacterial lipopolysaccharide (LPS), which signals through TLR4 and initiates the production of inflammatory cytokines, enhances 5-HT breakdown in the adult brain^29^. Despite data supporting a link between inflammation and serotonergic dysfunction, the capacity for PNS to influence this system during intrauterine development is largely unknown.

We hypothesized that an inflammatory response elicited by PNS is dependent upon maternal microbes and CCL2 signaling, and leads to altered serotonergic metabolism in the placenta and fetal brain, culminating in adverse behavioral outcomes in adult offspring. Through the use of our chronic restraint stress model in pregnant C57BL/6, GF, and CCL2^−/−^ mice, we demonstrate that PNS induces microbe-dependent intrauterine inflammation and placental serotonergic dysfunction, and that CCL2 is critical for mediating deficits in adult offspring behavior.

## Methods

### Animals and experimental design

**Fig. 1:**
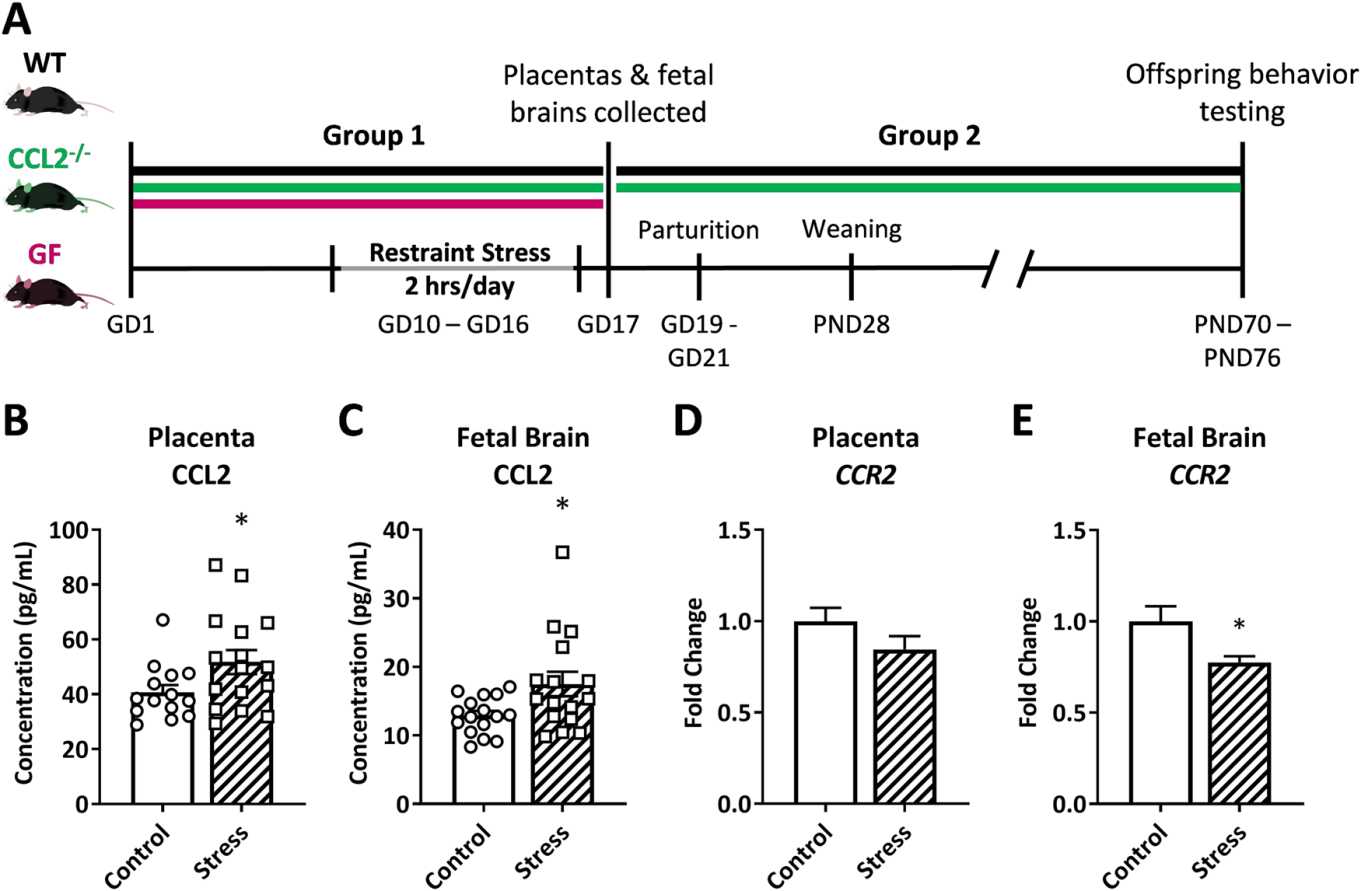
Prenatal stress leads to alterations in the CCL2–*CCR2* axis in the intrauterine environment. **a** WT, CCL2^−/−^, and GF pregnant mice were exposed to restraint stress for 2 h daily from GD10 to GD16. Placental and fetal brain tissue were collected from Group 1 animals on GD17. In Group 2, dams underwent parturition, and behavior of the WT and CCL2^−/−^ offspring was assessed at 10 weeks of age. CCL2 protein concentration in WT **b** placental (*n* = 14/7, 16/8 samples/litters in control, stress conditions) and **c** fetal brain (*n* = 16/5, 16/7 samples/litters in control, stress conditions) lysates. *CCR2* gene expression in WT **d** placentas (*n* = 15/9, 15/8 samples/litters in control, stress conditions) and **e** fetal brains (*n* = 14/9, 17/8 samples/litters in control, stress conditions). Bars represent mean ± SEM. *T* test: *p < 0.05. WT = wild type; GF = germ free; GD = gestational day.

Nulliparous 10-week old wild-type (WT) C57BL/6 mice and CCL2^−/−^ (knock-out; KO) mice were obtained from Jackson Laboratories (Bar Harbor, ME) and GF C57BL/6 mice from Taconic Biosciences (Germantown, NY) and maintained in conventional or sterile conditions at the Ohio State University. Mice were bred for 48 h, and copulation plugs were used to determine the first gestational day (GD1) of pregnancy. Pregnant dams were randomly assigned to stressed (PNS) or non-stressed groups. As previously described^4,5^, the PNS group underwent restraint stress from 9:00 a.m. to 11:00 a.m. on GD10–GD16 using a 50 mL conical tube with perforations to allow for ventilation, while the non-stressed group was left undisturbed. The timing of the restraint stress was chosen to coincide with key processes in neurodevelopment and the establishment of yolk sac placental circulation on E9–10^37,38^. In the first set of experiments, WT, CCL2^−/−^, and GF dams were euthanized on GD17 for tissue collection (Group 1). In a second set of experiments, WT and CCL2^−^^/−^ dams went through parturition and pups were weaned at PND 28, divided by sex, and co-housed with littermates, with a maximum of five mice per cage, until offspring behavioral testing was performed at 10 weeks of age by a person blinded to the experimental groups (Group 2; Fig. 1a). Four replicates using the same restraint stress paradigm were performed, with a total of 35 WT dams (16 control and 19 PNS), 34 CCL2^−^^/−^ dams (19 control and 15 PNS), and 11 GF dams (5 control and 6 PNS). Tissue was collected from, and behavior was performed, using a maximum of four offspring from each litter, balancing for sex. All experiments were conducted in accordance with the principles and procedures outlined in the National Institutes of Health Guidelines for the Care and Use of Experimental Animals and were approved by the Institutional Animal Care and Use Committee at the Ohio State University.

### Tissue collection

Dams were euthanized at GD17 using CO_2_ and a sterile cesarean section was performed to remove the uterus. Placentas and fetal brain tissue were excised, frozen on dry ice, and stored at −80 °C until processing. Fetal microdissection and examination of the reproductive structures were used to determine fetal sex, which was confirmed using genotyping of fetal tails for the SRY gene.

### Quantitative real-time PCR

Trizol reagent (Invitrogen, Carlsbad, CA) was used for RNA extraction and cDNA was synthesized using the High-Capacity cDNA Reverse Transcription Kit (Applied Biosystems, Foster City, CA), per manufacturer’s instructions. PCR reactions were performed using the Taqman Gene Expression Master Mix protocol (Applied Biosystems, Foster City, CA) and QuantStudio 3 Real-Time PCR Systems machine (Applied Biosystems, Foster City, CA). Genes of interest are listed in Supplementary Table 1. qPCR data is presented as fold change compared with the non-stressed group, using the 2^−ΔΔCt^ method. For comparing across WT, CCL2^−/−^, and GF tissue, data were normalized to the WT control group. For determining sex differences, data were compared with the female control group. *SDHA* and *TBP* were used as endogenous control genes for placental samples, and *GAPDH* was used for fetal brain samples.

### ELISA

Placentas and fetal brains were homogenized in T-PER Tissue Protein Extraction Reagent (Thermo Scientific, Waltham, MA) by sonication. CCL2 was measured in the protein lysates using the Mouse CCL2/JE/MCP-1 Duoset ELISA (R&D Systems, Minneapolis, MN).

### LC/MS

The Mass Spectrometry and Proteomics Facility at the Ohio State University extracted metabolites from placental and fetal brain tissues and determined the concentration of tryptophan, serotonin, and 5-HIAA. Concentrations were normalized to tissue weights.

### Social behavior testing

Sociability was assessed using the three-chamber social behavior test, as previously described^4^. Briefly, testing was completed in a three-chamber box with metal cages placed in the center of the two end chambers. A 10-min acclimation phase was followed by a 10-min test phase, during which a novel object (orange Pyrex cap (Corning, NY)) and a social stimulus DBA2J mouse of the same sex and age (Jackson Laboratories, Bar Harbor, ME) were placed in the metal cages. The chamber that contained the conspecific mouse was randomly assigned for each test mouse to control for side preference. The trials were recorded using Noldus EthoVision software (Wageningen, The Netherlands). Social approach behavior was calculated by subtracting the time spent actively investigating the novel object from the time spent with the social stimulus mouse and dividing by the total time interacting with both cages.

### Light–dark preference test

Light–dark preference testing was performed in a 40 × 40 × 25 cm Plexiglas box divided into equal 20 × 40 × 25 cm compartments by a black Plexiglass separator, with a 3 × 10 cm doorway allowing for the test mouse to transition between zones. The light compartment was illuminated at an intensity of 150 lux, while the dark compartment was enclosed by a black Plexiglass cover. The test mouse was placed into the light compartment to begin the 5 min trial, and time to enter the dark compartment, duration of time spent in each zone, and total distance traveled was measured using Fusion software (Omnitech Electronics, Inc., Columbus, OH).

### Statistical analyses

Statistical tests were performed using Graphpad Prism. Multifactorial analysis of variance (ANOVA) followed by a Tukey’s post hoc test was performed to determine possible effects of sex. For tests in which the main effect of sex and the interaction between sex and stress were not significant, data were collapsed and two-tailed unpaired *t*-tests were used to determine statistical significance between stressed and non-stressed groups, and unpaired *t* tests with Welch’s correction were performed for groups with statistically significantly different variances (F test). Differences between WT, CCL2^−/−^, and GF tissue were assessed using a two-way ANOVA followed by a Tukey’s post hoc test. The ROUT method was used to identify outliers (with Q set to 1%), which were excluded from analyses. Sample sizes were chosen based on a power analysis using values from prior experiments^5^. Significance was defined as *p* < 0.05.

## Results

### PNS leads to changes in the CCL2–*CCR2* axis in the intrauterine environment

PNS increased CCL2 protein levels in the placenta (Fig. 1b; *t*(24.36) = 2.15; *p* = 0.042) and in the fetal brain (Fig. 1c; *t*(19.22) = 2.40; *p* = 0.027). Expression of C-C Motif Chemokine Receptor 2 (*CCR2*), the primary receptor for CCL2, was not altered in placentas (Fig. 1d; *t*(28) = 1.49; *p* = 0.148), but was decreased in PNS fetal brains (Fig. 1e; *t*(17.09) = 2.49; *p* = 0.023). Together, this suggests that our model of PNS results in disruption of the CCL2–*CCR2* axis in the intrauterine environment.

### PNS increases fetal brain *IL6* in a CCL2-dependent manner

**Fig. 2:**
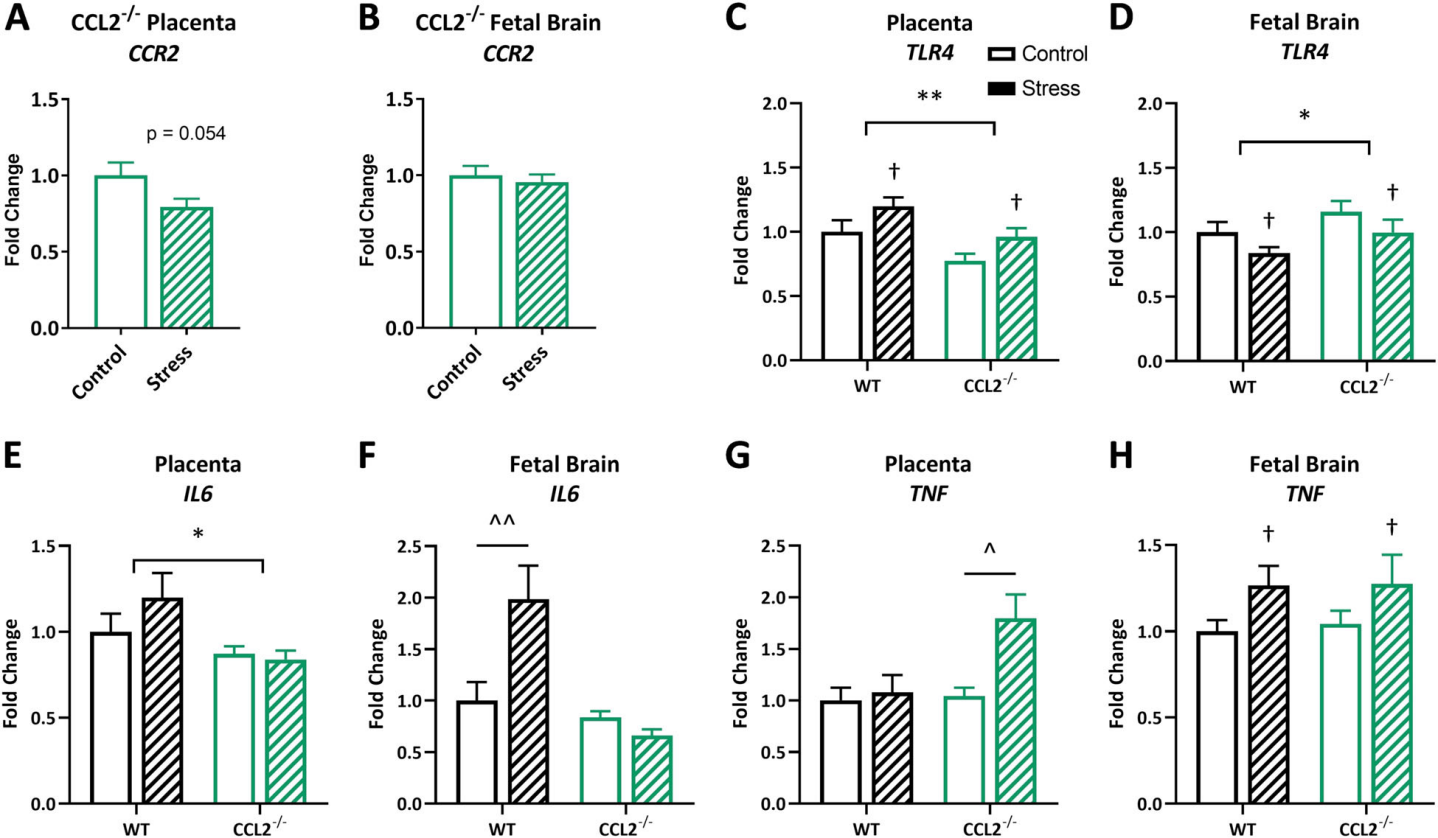
Prenatal stress increases fetal brain *IL6* in a CCL2-dependent manner. *CCR2* gene expression in CCL2^−/−^**a** placentas (*n* = 13/7, 13/5 samples/litters in control, stress conditions) and **b** fetal brains (*n* = 16/8, 13/5 samples/litters in control, stress conditions) following stress. Gene expression of the pattern recognition receptor *TLR4* in the WT and CCL2^−/−^ placenta (*n* = 15/8, 16/8, 14/8, 13/5 samples/litters in WT control, WT stress, CCL2^−/−^ control, CCL2^−/−^ stress conditions) (**c**) and fetal brain (*n* = 14/8, 18/8, 15/8, 13/5 samples/litters in WT control, WT stress, CCL2^−/−^ control, CCL2^−/−^ stress conditions) (**d**). Gene expression of pro-inflammatory cytokines **e, f***IL6* and **g, h***TNF* in the WT and CCL2^−/−^ placenta (*IL6*: *n* = 16/9, 14/8, 14/8, 13/5; *TNF*: *n* = 16/9, 16/8, 14/8, 13/5 samples/litters in WT control, WT stress, CCL2^−/−^ control, CCL2^−/^^−^ stress conditions) and fetal brain (*IL6*: *n* = 14/9, 15/7, 16/8, 12/5; *TNF*: *n* = 15/9, 14/8, 16/8, 13/5 samples/litters in WT control, WT stress, CCL2^−/−^ control, CCL2^−/−^ stress conditions), respectively. Bars represent mean ± SEM. Two-way ANOVA: asterisks (*) represent main effect of genotype (*p < 0.05; **p < 0.01); daggers (†) represent main effect of stress (†*p* < 0.05); carets (^) represent stress × genotype interaction with the significant Tukey post hoc test (^*p* < 0.05; ^^*p* < 0.01).

To determine the role of CCL2 in the intrauterine environment following PNS, CCL2^−/−^ placentas and fetal brains were examined and compared with WT. Absence of CCL2 in CCL2^−/−^ tissues was confirmed by PCR. Exposure to PNS resulted in a nonsignificant decrease in *CCR2* expression in the CCL2^−/−^ placenta (Fig. 2a; *t*(24) = 2.02; *p* = 0.054), while there was no change in the fetal brain (Fig. 2b; *t*(27) = 0.53; *p* = 0.599). If maternal microbial homeostasis is disrupted by PNS, microbes or their products may reach the intrauterine environment, which can be monitored in part through the expression of pattern recognition receptors, such as TLR4. PNS increased *TLR4* expression in the placenta (Fig. 2c; main effect of stress: *f*(1,54) = 6.97; *p* = 0.011) and decreased *TLR4* in the fetal brain (Fig. 2d; main effect of stress: *f*(1,56) = 4.48; *p* = 0.039), though CCL2^−/−^ placentas had lower expression (Fig. 2c; main effect of genotype: *f*(1,54) = 10.19; *p* = 0.002) and CCL2^−/−^ fetal brains had higher expression (Fig. 2d; main effect of genotype: *f*(1,56) = 4.35; *p* = 0.042) compared with WT.

Since TLR4 activation can enhance expression of pro-inflammatory cytokines, a panel of cytokines and immune-related receptors was examined in the WT placenta and fetal brain (Supplementary Table 2). Among the genes of interest, the most compelling stress-induced immune dysregulation was evident in the intrauterine expression of *IL6* and *TNF* following PNS. CCL2^−/−^ placentas had decreased expression of *IL6* compared with WT (Fig. 2e; main effect of genotype: *f*(1,53) = 6.18; *p* = 0.016), while there was a stress × genotype interaction in the fetal brain (Fig. 2f; *f*(1,53) = 8.50; *p* = 0.005), with stress increasing *IL6* expression only in WT mice. These data indicate that PNS increases fetal brain *IL6* expression in a CCL2-dependent manner. Notably, there was a sex difference in CCL2^−^^/−^ fetal brain *IL6* expression, with males expressing lower levels compared with females (Supplementary Fig. 1a; main effect of sex: *f*(1,24) = 4.55; *p* = 0.043), and a stress-induced decrease in both sexes (main effect of stress: *f*(1,24) = 5.27; *p* = 0.031). Interestingly, PNS increased *TNF* expression in CCL2^−/−^ placentas, but not WT (Fig. 2g; stress × genotype interaction: *f*(1,55) = 4.61; *p* = 0.036), and in both WT and CCL2^−/−^ fetal brains (Fig. 2h; main effect of stress: *f*(1,54) = 5.29; *p* = 0.025).

Altogether, these data suggest that the placenta may be responding to a microbe-associated molecular pattern (possibly a TLR4 ligand) following PNS, leading to inflammation within the fetal brain due to CCL2 signaling.

### In the absence of microbes, PNS does not induce intrauterine inflammation

**Fig. 3:**
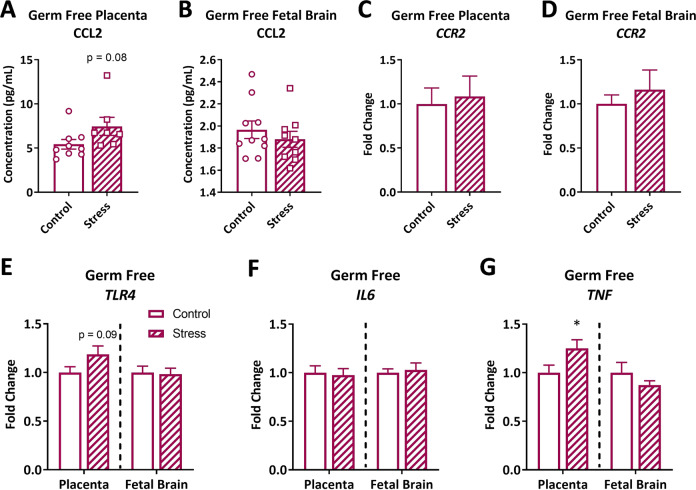
In the absence of microbes, prenatal stress does not induce intrauterine inflammation. CCL2 protein concentration in GF **a** placental (*n* = 9/5, 7/5 samples/litters in control, stress conditions) and **b** fetal brain lysates (*n* = 10/5, 9/5 samples/litters in control, stress conditions). *CCR2* gene expression in the **c** placenta (*n* = 13/5, 14/6 samples/litters in control, stress conditions) and **d** fetal brain (*n* = 11/5, 12/6 samples/litters in control, stress conditions) following stress in GF dams. **e***TLR4* gene expression in the GF placenta and fetal brain (placenta: *n* = 13/5, 14/6; fetal brain: *n* = 12/5, 14/6 samples/litters in control, stress conditions). Gene expression of pro-inflammatory cytokines **f***IL6* (placenta: 12/5, 13/6; fetal brain: *n* = 11/5, 14/6 samples/litters in control, stress conditions) and **g***TNF* (placenta*:n* = 12/5, 14/6; fetal brain: *n* = 12/5, 13/6 samples/litters in control, stress conditions) in the GF placenta and fetal brain. Dashed lines separate placental and fetal brain gene expression data, which were analyzed separately. Bars represent mean ± SEM. *T* test: *p < 0.05.

In GF animals, PNS resulted in a nonsignificant increase in CCL2 protein in the placenta (Fig. 3a; *t*(14) = 1.87; *p* = 0.083), and no change in the fetal brain (Fig. 3b; *t*(17) = 0.80; *p* = 0.433). *CCR2* gene expression in GF tissue was not altered by PNS (placenta: Fig. 3c; *t*(25) = 0.28; *p* = 0.778; fetal brains Fig. 3d; *t*(15.36) = 0.66; *p* = 0.522). Likewise, *TLR4* gene expression in GF tissue remained unchanged (Fig. 3e; placenta: *t*(25) = 1.77; *p* = 0.089; fetal brain: *t*(24) = 0.19; *p* = 0.85). Finally, *IL6* did not differ in GF tissues (Fig. 3f; placenta: *t*(23) = 0.26; *p* = 0.800; fetal brain: *t*(19.65 = 0.34; *p* = 0.741), while only GF placental *TNF* was altered by PNS (Fig. 3g; placenta: *t*(24) = 2.07; *p* = 0.049; fetal brain: t(14.56) = 1.10; *p* = 0.288). Together, this suggests that PNS fails to initiate an inflammatory cascade within the fetal brain when microbes are not present.

### Intrauterine tryptophan and serotonin availability is partially dependent upon CCL2 and microbes

**Fig. 4:**
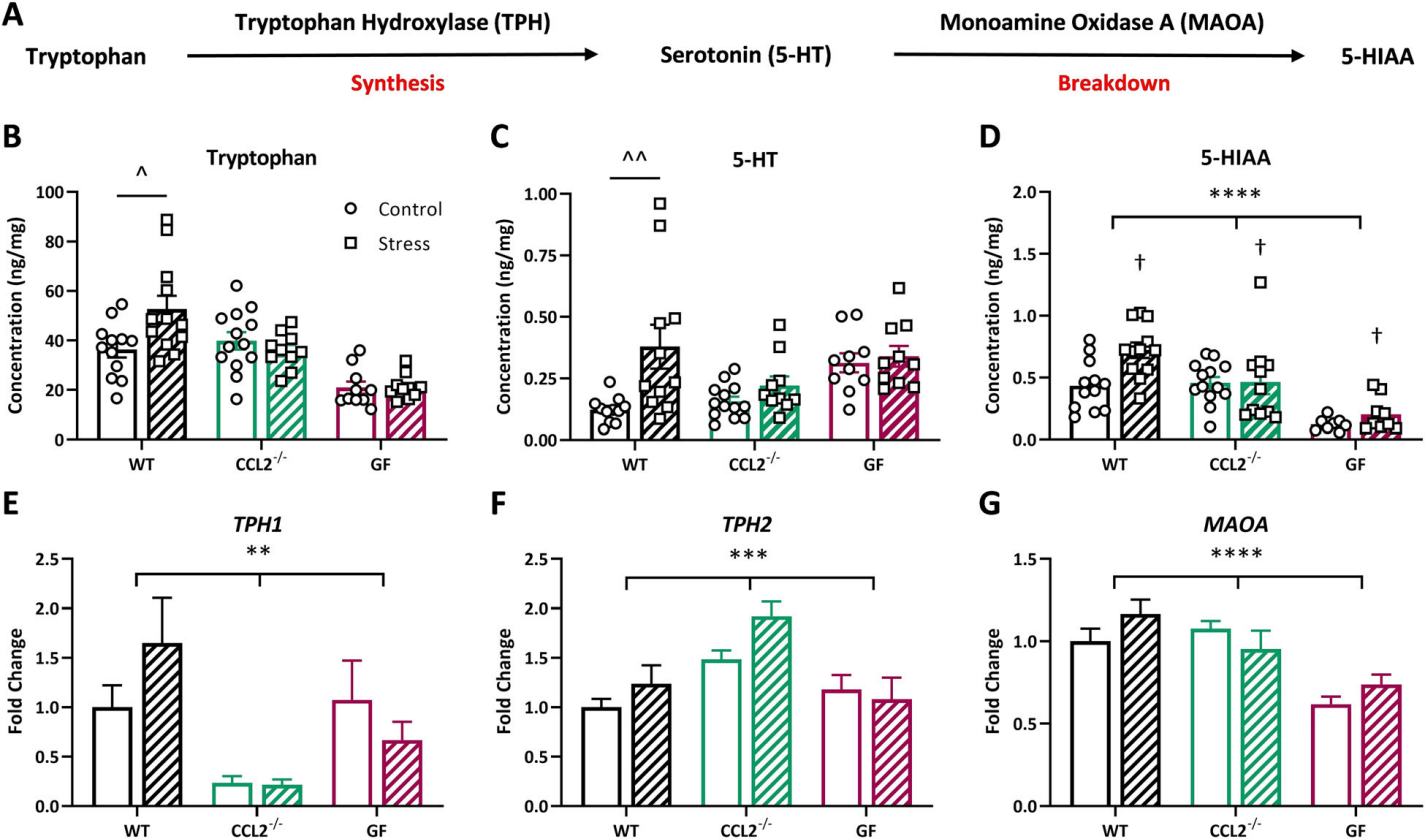
Placental tryptophan and serotonin availability is dependent upon CCL2 and microbes. **a** Tryptophan hydroxylase (TPH) metabolizes tryptophan to serotonin, which is then broken down into 5-HIAA by monoamine oxidase A (MAOA). **b** Concentration of tryptophan in WT (*n* = 12/7, 12/7 samples/litters in control, stress conditions), CCL2^−/−^ (*n* = 13/7, 10/5 samples/litters in control, stress conditions), and GF (*n* = 10/5, 10/6 samples/litters in control, stress conditions) mice following exposure to restraint stress. **c** Concentration of 5-HT in WT (*n* = 11/6, 11/7 samples/litters in control, stress conditions), CCL2^−/−^ (*n* = 13/7, 10/5 samples/litters in control, stress conditions), and GF (*n* = 10/5, 10/6 samples/litters in control, stress conditions) placentas. **d** Concentration of 5-HIAA in WT (*n* = 12/7, 12/7 samples/litters in control, stress conditions), CCL2^−/−^ (*n* = 13/7, 11/5 samples/litters in control, stress conditions), and GF (*n* = 8/5, 9/6 samples/litters in control, stress conditions) placentas. Expression of **e***TPH1* (*n* = 16/9, 14/8, 11/7, 8/5, 13/5, 14/6 samples/litters in WT control, WT stress, CCL2^−/−^ control, CCL2^−/−^ stress, GF control, GF stress conditions) and **f***TPH2* (*n* = 14/9, 15/8, 10/4 12/5, 10/5, 9/6 samples/litters in WT control, WT stress, CCL2^−/−^ control, CCL2^−/−^ stress, GF control, GF stress conditions), two isoforms of TPH present in the placenta. **g***MAOA* gene expression (*n* = 16/9, 16/8, 14/8, 13/5, 13/5, 14/6 samples/litters in WT control, WT stress, CCL2^−/−^ control, CCL2^−/−^ stress, GF control, GF stress conditions). Bars represent mean ± SEM. Two-way ANOVA: asterisks (*) represent main effect of genotype (**p < 0.01; ***p < 0.001; ****p < 0.0001); daggers (†) represent main effect of stress (†*p* < 0.05); carets (^) represent stress × genotype interaction with the significant Tukey post hoc test (^*p* < 0.05; ^^*p* < 0.01).

Given previous data from our lab indicating altered 5-HT concentrations following PNS exposure^4^, our aim was to determine if these alterations have intrauterine origins. The pathway through which 5-HT is synthesized and broken down is depicted in Fig. 4a. PNS increased levels of placental tryptophan and 5-HT in WT mice, but not CCL2^−/−^ or GF mice (Fig. 4b, c; tryptophan: stress × genotype interaction: *f*(2,61) = 4.92; *p* = 0.011; 5-HT: stress × genotype interaction: *f*(2,59) = 3.48; *p* = 0.037). Tryptophan concentration control GF placentas were lower than both WT (adjusted *p* = 0.046) and CCL2^−/−^ (adjusted *p* = 0.004), and there was a significant stress × sex interaction on GF placental tryptophan levels (Supplementary Fig. 1b; stress × sex interaction: *f*(1,16) = 10.69; *p* = 0.005) that was absent in WT or KO tissue. In contrast, there were main effects of stress and genotype on 5-HIAA concentrations (Fig. 4d; stress: *f*(1,59) = 5.70; *p* = 0.020; genotype: *f*(2,59) = 20.58; *p* < 0.0001), but no stress × genotype interaction.

In order to determine whether PNS alters 5-HT synthesis and breakdown, gene expression of the enzymes involved in synthesizing and metabolizing 5-HT were investigated. Since the placenta has been shown to express two isoforms of TPH (TPH1 and TPH2)^39^, both were examined. There was a main effect of genotype (but not PNS) on placental *TPH1* expression (Fig. 4e; *f*(2,70) = 5.93; *p* = 0.004), with considerably lower expression in the CCL2^−/−^ placenta compared with WT and GF. There was also a main effect of genotype on placental *TPH2* expression (Fig. 4f; *f*(2,64) = 9.09; *p* = 0.003), with the highest expression now in CCL2^−/−^ compared with WT and GF tissue. There was also a main effect of genotype on placental *MAOA* expression (Fig. 4e; *f*(2,80) = 16.27; *p* < 0.0001), with decreased levels in GF tissue compared with WT and CCL2^−/−^.

Of note, these changes appear to be restricted to the placenta, as PNS had no effect on tryptophan levels (*t*(22) = 0.009; *p* = 0.993), *TPH2* expression (*t*(30) = 0.60; *p* = 0.554), 5-HT concentrations (*t*(22) = 0.47; *p* = 0.639), *MAOA* expression (*t*(31) = 1.24; *p* = 0.224), or 5-HIAA levels (*t*(22) = 0.003; *p* = 0.998) in the WT fetal brain (Supplementary Fig. 2a–e), and thus these outputs were not examined in CCL2^−/−^ or GF fetal brain tissue. Together, our data suggest that PNS increases placental tryptophan and 5-HT availability in a manner that is at least partly dependent upon CCL2 and microbes.

### PNS leads to CCL2-dependent behavioral abnormalities in adult offspring

**Fig. 5:**
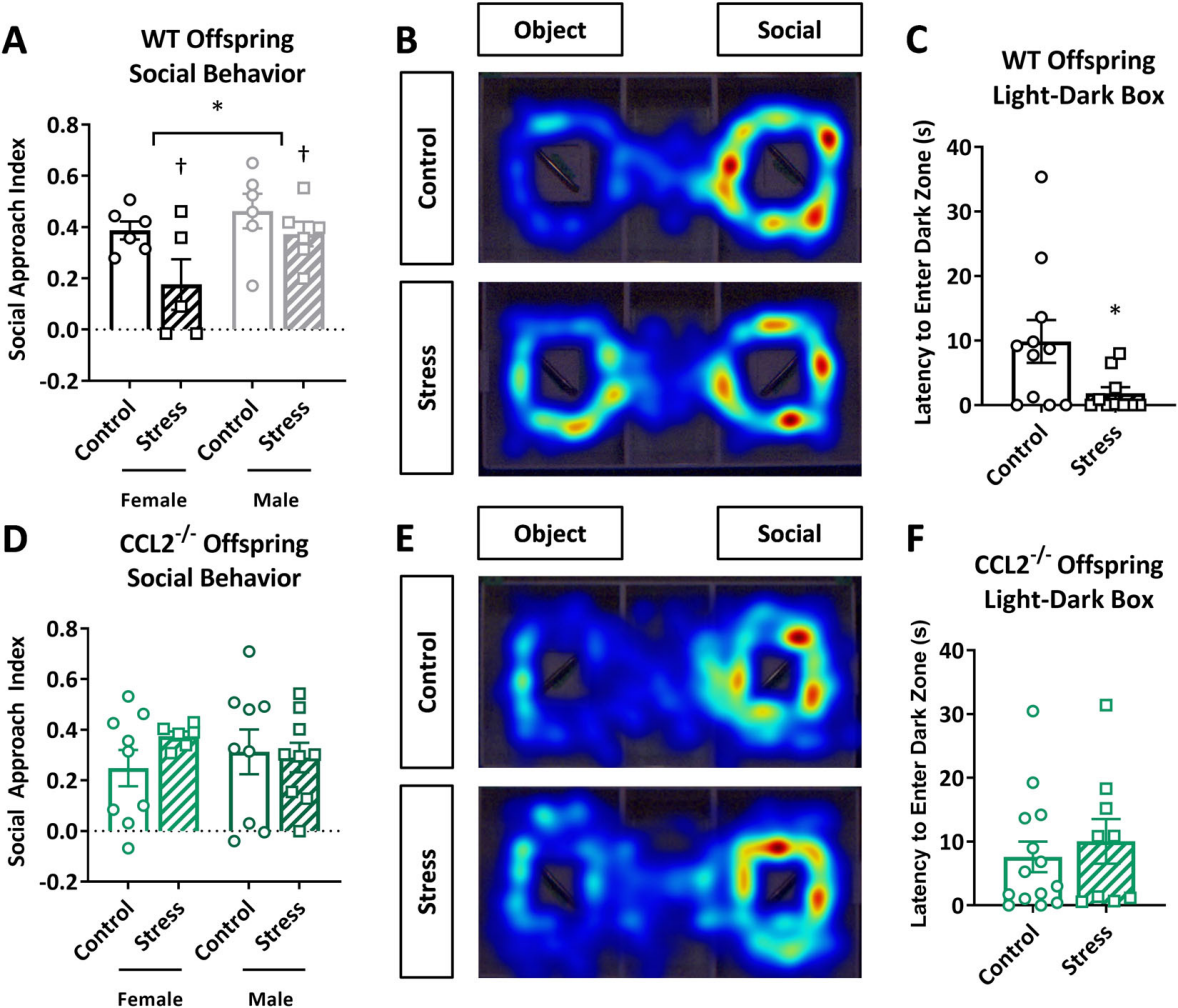
Prenatal stress leads to CCL2-dependent behavioral abnormalities in adult offspring. WT offspring: **a** social approach index in the three-chamber social behavior test (*n* = 6, 5, 6, 6 mice in female control, female stress, male control, male stress conditions); **b** representative heat maps of time spent exploring the three chambers in the social behavior test; **c** latency to enter the dark chamber of the light–dark box (*n* = 11, 10 mice in control, stress conditions). CCL2^−/−^ offspring: **d** social approach index (*n* = 9, 6, 9, 9 mice in female control, female stress, male control, male stress conditions); **e** representative heat maps of the social behavior test; **f** latency to enter the dark chamber of the light–dark box (*n* = 13, 9 mice in control, stress conditions). Bars represent mean ± SEM. *T* test: *p < 0.05. Two-way ANOVA: asterisks (*) represent main effect of sex (*p < 0.05); daggers (†) represent main effect of stress (†*p* < 0.05).

WT PNS offspring exhibited deficits in social approach behavior (Fig. 5a; main effect of stress: *f*(1,19) = 5.61; *p* = 0.029, also reflected in representative heat maps, Fig. 5b) that were sex-dependent (main effect of sex: *f*(1,19) = 4.60; *p* = 0.045). In the light–dark preference test, PNS offspring had decreased latency to enter the dark zone (Fig. 5c; *t*(11.62) = 2.34; *p* = 0.038). Stress did not alter locomotor activity (*t*(13.80) = 0.09; *p* = 0.930) or initial side-preference within the social apparatus (*t*(21) = 0.33; *p* = 0.744; Supplementary Fig. 3a, b). While PNS did not impact duration of time in the light compartment (Supplementary Fig. 3c; *t*(23) = 0.62; *p* = 0.539) or locomotor activity in the light–dark preference test, female mice traveled a greater distance compared with male mice during testing (Supplementary Fig. 3d; main effect of sex: *f*(1,21) = 15.08; *p* = 0.0009).

Notably, CCL2^−/−^ offspring were protected from social deficits, as measured by the social approach index (Fig. 5d, e; stress effect: *f*(1,29) = 0.54; *p* = 0.470) and did not display sex differences (sex effect: *f*(1,26) = 0.02; *p* = 0.883). Similarly, CCL2^−/−^ offspring did not differ in their latency to enter the dark zone during light–dark preference testing (Fig. 5f; *t*(21) = 0.59; *p* = 0.560). Although CCL2^−/−^ locomotor activity did not differ due to PNS in the social apparatus (Supplementary Fig. 3e; *t*(32) = 0.64; *p* = 0.529), there was an initial side-preference (Supplementary Fig. 3f; main effect of sex: *f*(1,30) = 8.04; *p* = 0.008; main effect of stress: *f*(1,30) = 8.27; *p* = 0.007), which was addressed by randomization of the side in which the conspecific mouse was placed and by randomization of the order in which male and female offspring were tested. Duration spent in the light (Supplementary Fig. 3g; *t*(30) = 0.34; *p* = 0.734) and distance traveled (Supplementary Fig. 3h; *t*(21.84) = 0.78; *p* = 0.441) were not impacted by PNS or sex during light–dark testing. Together, our data indicate that PNS results in social- and anxiety-related behavioral abnormalities in the offspring, which are ameliorated in the absence of CCL2.

## Discussion

We provide evidence that maternal microbes and the chemokine CCL2 play critical roles in mediating the sequelae of PNS, including intrauterine inflammatory and serotonergic dysfunction, as well as offspring behavioral abnormalities. This is the first time, to our knowledge, that maternal microbes and CCL2 have been linked to intrauterine inflammation and placental serotonin availability and that CCL2 has been implicated in mediating offspring behavioral deficits following PNS.

Recent clinical and preclinical studies suggest that PNS leads to inflammation in the intrauterine environment^5,6,20,40,41^. To extend these findings, we demonstrate that PNS increases CCL2 protein levels in the placenta and fetal brain. As a chemokine, CCL2 recruits circulating CCR2+ monocytes to infiltrate sites of active inflammation and mature into tissue macrophages or produce cytokines^21^. Thus, the reduced expression of *CCR2* in the fetal brain could be due to a compensatory mechanism to counteract detrimental elevations in CCL2. As expected, a decrease in fetal brain *CCR2* expression following PNS was not present in CCL2^−/−^ mice.

Previously, our lab has shown that restraint stress alters the maternal gut microbiome^5^. Stress can increase permeability of the gut epithelial barrier, allowing microbes or microbial components to escape from the gut lumen and enter the bloodstream^42–44^. The presence of microbes in the intrauterine environment is highly controversial^45–47^, and was not investigated in the present study. Instead, GF tissues were examined to ascertain the necessity of maternal microbes in mediating the immunomodulatory effects of PNS in the intrauterine environment. To our knowledge, this study is the first to examine the GF intrauterine environment in the context of PNS, though prior studies have shown that GF mice exhibit aberrant behavior^48,49^ and an exaggerated stress response^50^. While placental CCL2 tended to increase in PNS GF animals, the GF fetal brain appeared to be protected from inflammatory signaling, with no change in CCL2 protein or *CCR2* expression.

We next investigated the contribution of CCL2 and maternal microbes to PNS-induced inflammation in the intrauterine environment. Even if viable microbes are not present in the placenta, microbial components in the maternal bloodstream can initiate an immune response within the uterus by signaling through TLRs^51^; thus, we investigated bacterial LPS-receptor TLR4 as a potential mediator of stress-induced inflammation. In our model, restraint stress increased *TLR4* gene expression in both WT and CCL2^−/−^ placentas, and decreased expression in the fetal brain, suggesting that microbes or microbial components may be reaching the placenta and initiating an immune response. Furthermore, these data indicate that PNS-induced increases in *TLR4* are independent of CCL2 production in the intrauterine environment, despite lower *TLR4* expression in CCL2^−/−^ placentas at baseline. Of note, PNS did not induce significant changes in placental or fetal brain *TLR4* expression under GF conditions.

In addition to the alterations in *TLR4* expression, cytokine expression was also dysregulated in the intrauterine environment following exposure to PNS. Specifically, stress enhanced expression of the pro-inflammatory cytokine *IL6* in the WT fetal brain, which was ameliorated in the CCL2^−/−^ fetal brain. However, *TNF* expression was elevated in both WT and CCL2^−/−^ fetal brains, suggesting that IL-6 production, in particular, might be dependent upon CCL2 signaling. These data are in line with previous reports demonstrating that recombinant CCL2 administered directly into the brain increases production of IL-6, but not TNF-α^52^. Overall, we theorize that PNS enhances CCL2 secretion in the fetal brain, which is necessary for *IL6* upregulation. PNS did not alter fetal brain *IL6* or *TNF* expression under GF conditions, suggesting that PNS does not induce fetal neuroimmune activation in the absence of maternal microbes. Since CCL2 levels are lower in GF mice compared with WT mice at baseline^53^, our findings further support the idea that PNS increases *IL6* expression in the fetal brain in normal CCL2 conditions, but fails to do so when CCL2 is low or absent.

In contrast to the fetal brains, PNS did not alter placental *IL6* expression in WT or CCL2^−/−^ mice. However, PNS increased *TNF* expression in CCL2^−/−^ placentas, but not in WT. Interestingly, in the absence of microbes, PNS also elevated expression of *TNF* in the placenta. Together, this may indicate that CCL2 suppresses placental *TNF* expression following PNS, which is distinct from the function of CCL2 in the developing brain. This is further consistent with the finding that administration of LPS to CCL2^−/−^ mice increases serum TNF-α to a greater degree than in WT mice^54,55^, indicating that CCL2 may be suppressing or modulating TNF-α production in the face of an immune challenge.

One consideration in using GF and CCL2^−/−^ mice is that their immune systems develop in a dramatically different fashion compared with WT mice. Indeed, exposure to commensal microbes is necessary for education and priming of immune cells, and GF mice have been shown to have deficits in function of both the innate and adaptive immune system^56,57^. However, there is evidence that GF mice are capable of producing IL-6 in response to restraint stress similar to WT mice^58^, suggesting that the absence of *IL6* upregulation in GF fetal brains cannot be explained by baseline developmental differences in IL-6 production. On the other hand, CCL2^−/−^ mice have impairments in recruiting monocytes to sites of infection, though the number of circulating leukocytes is similar to WT mice^59^. In our model, we observed slight but significant decreases in *IL6* expression in CCL2^−/−^ tissue compared with WT at baseline, but not *TNF*. Others have also shown that CCL2^−/−^ mice are capable of mounting measurable, albeit blunted, neuroimmune responses to a peripheral immune challenge^54^, and that astrocytes from CCL2^−/−^ brains are capable of increasing production of IL-6 and TNF in response to in vitro challenge^60^. These in vivo and in vitro responses are easily differentiated from baseline differences due to KO status, suggesting that the PNS-induced alterations in cytokine expression observed in our model cannot be attributed solely to developmental differences.

The presence of inflammatory cytokines in the brain can adversely impact neurodevelopment through various mechanisms, such as direct neuronal injury^61,62^, overall disruption of neuroimmune function^28^, and/or altered serotonergic metabolism^29,35,63^. We previously found that our model of maternal restraint stress leads to elevated levels of 5-HT in the plasma of male offspring that also exhibited decreased sociability^4^. 5-HT is a monoamine neurotransmitter that plays a key role in maintaining a healthy pregnancy, in addition to its role as a neurotrophic factor in the developing brain^33,64^. Indeed, high levels of placental serotonin have been associated with adverse outcomes in pregnancy, including intrauterine growth restriction^65^ and preeclampsia^66^, which have both been linked to neurodevelopmental disorders^67,68^. In our model, PNS increased concentrations of tryptophan and 5-HT in WT placentas, which was ameliorated in both CCL2^−/−^ and GF placentas, indicating a key role of CCL2 and maternal microbes in modulating tryptophan and 5-HT availability. The lack of a stress effect on *TPH1*, *TPH2*, and *MAOA* expression further suggests that stress modulates *availability* of tryptophan and 5-HT rather than synthesis or breakdown. PNS also increased 5-HIAA concentrations; however, this elevation was evident regardless of CCL2 and GF status, suggesting that modifications to 5-HIAA may be independent of CCL2 and microbes. Additionally, the low levels of *MAOA* and 5-HIAA in GF placentas suggests that 5-HT breakdown is in-part dependent on microbes. Interestingly, PNS did not impact serotonin in the fetal brain; instead, our data suggest that PNS may mediate behavioral outcomes indirectly through imbalances in placental serotonin concentrations. Indeed, elevated levels of serotonin in the placenta can disrupt the development of serotonergic neurons in the fetal brain^35^, which presents an area for further investigation in this model. Finally, although our gene expression data suggest that PNS does not impact the enzymes involved in synthesizing and metabolizing 5-HT, we cannot rule out functional changes in the enzymes.

PNS is associated with anxiety- and depressive-like behaviors in offspring, and deficits in social interaction characteristic of autism spectrum disorders; this is evident both in rodent models^4–8,69,70^ and in humans^71,72^. Here, we show that PNS indeed leads to altered social and anxiety-like behaviors in adult offspring, which aligns with our previous findings^4,5^. Furthermore, our current dataset suggests that offspring behavioral deficits can manifest regardless of sex, though male offspring demonstrated greater preference for social stimuli compared with female offspring. The etiology of behavioral disruption is complex, often case-specific, and influenced by a multitude of factors. One such factor is exposure to elevated cytokines, particularly IL-6, during gestation^73,74^. Administration of IL-6 to pregnant mice induces offspring anxiety-like behavior^75^, and deletion of IL-6 receptor in trophoblast cells ameliorates deficits in sociability due to maternal inflammation^76^. Since PNS increased fetal brain IL-6, and this was mitigated in the absence of CCL2, we hypothesized that CCL2 would play an indirect role in regulating the development of aberrant behavior through the modification of IL-6. Indeed, the sociability deficits and anxiety-like behavior observed in WT adult offspring were ameliorated in CCL2^−/−^ offspring. While the use of a global CCL2 KO animal in the present study indicates the necessity of CCL2 in disrupting the development of certain behavioral circuits, the exact timing and mechanism by which CCL2 acts prenatally still needs to be investigated. Conclusions about the role of microbes in behavioral regulation are limited by the baseline aberrant behavior observed GF mice, including social deficits and anxiolytic behavior^48,77–79^. Careful and meticulous dissection of behavioral outcomes in GF offspring due to PNS, not explored in the current study, represents an avenue for future investigation.

Despite the prevalence of stress in modern day society, and the deleterious effects of maternal stress exposure on neurodevelopment, options for treatment or prophylaxis are suboptimal. Here, we provide evidence of the critical role of microbes and CCL2 in mediating fetal brain inflammation and placental tryptophan and 5-HT availability following maternal stress and leading to aberrant sociability and anxiety-like behavior in adult offspring. Sex differences among certain parameters, but not all, indicate that vulnerability to PNS is partially sex-biased. Altogether, these data suggest that the sequelae of maternal stress have prenatal origins, and that maternal microbes and CCL2 are tantalizing targets for developing novel treatments.

## Supplementary information

Supplementary information

Supplementary Figure 1

Supplementary Figure 2

Supplementary Figure 3

Supplementary Tables
