# Supplementary material for: Prenatal stress causes intrauterine inflammation and serotonergic dysfunction, and long-term behavioral deficits through microbe- and CCL2-dependent mechanisms
Source: Transl Psychiatry. 2020 Jun 16;10:191. doi: 10.1038/s41398-020-00876-5 (PMC7297973; doi:10.1038/s41398-020-00876-5)
Supplement: Supplementary file 1 — Supplementary information [file 41398_2020_876_MOESM1_ESM.docx]

**Prenatal Stress Causes Intrauterine Inflammation and Serotonergic Dysfunction, and Long-Term Behavioral Deficits Through Microbe- and CCL2-Dependent Mechanisms**

Supplementary Information

**Supplementary Figure Legends**

**Supplementary Figure 1: Intrauterine Sex Differences.** (A) Gene expression of pro-inflammatory cytokine *IL6* in female and male CCL2^-/-^ fetal brains (n = 9/4, 7/4, 7/5, 5/5 samples/litters in female control, female stress, male control, male stress conditions) following prenatal stress. (B) Concentration of tryptophan in female and male GF placentas (n = 5/3, 5/5, 5/4, 5/4 samples/litters in female control, female stress, male control, male stress conditions). Bars represent mean ± SEM. Two-way ANOVA: asterisks (*) represent main effect of sex (* p < 0.05); daggers (†) represent main effect of stress († p < 0.05); carets (^) represent stress x genotype interaction with significant Tukey post hoc test (^ p < 0.05).

**Supplementary Figure 2: Effect of Prenatal Stress on Serotonin Metabolism in Fetal Brains.** (A) Concentrations of tryptophan (n = 12/7, 12/7 samples/litters in control, stress conditions) in the WT fetal brain following exposure to restraint stress. (B) *TPH2*, the isoform of TPH present in neuronal tissue, gene expression in the WT fetal brain (n = 14/9, 18/8 samples/litters in control, stress conditions). (C) Concentrations of 5-HT (n = 12/7, 12/7 samples/litters in control, stress conditions) in the WT fetal brain. (D) *MAOA* gene expression in the WT fetal brain (n = 15/9, 18/8 samples/litters in control, stress conditions). (E) Concentrations of 5-HIAA in the WT fetal brain (n = 12/7, 12/7 samples/litters in control, stress conditions). Bars represent mean ± SEM.

**Supplementary Figure 3:** **Effect of Prenatal Stress on Behavioral Characteristics.** (A) Distance traveled in the social behavior apparatus during the acclimation and test phases (n = 12, 11 mice in control, stress conditions). (B) Initial side preference in the acclimation phase of the social behavior test (n = 12, 11 mice in control, stress conditions). (C) Duration of time spent in the light zone (n = 12, 13 mice in control, stress conditions) and (D) distance traveled in the light dark preference test (n = 6, 7, 6, 6 mice in female control, female stress, male control, male stress conditions) in WT offspring. (E) Distance traveled in the social behavior apparatus during the acclimation and test phases for CCL2^-/-^ offspring (n = 18, 16 mice in control, stress conditions). (F) Initial side preference in the acclimation phase of the social behavior test for CCL2^-/-^ offspring (n = 9, 7, 9, 9 mice in female control, female stress, male control, male stress conditions). (G) Duration of time spent in the light zone (n = 17, 15 mice in control, stress conditions) and (H) distance traveled in the light-dark preference test (n = 16, 11 mice in control, stress conditions) for CCL2^-/-^ offspring. Bars represent mean ± SEM. Two-way ANOVA: asterisks (*) represent main effect of sex (** p < 0.01; *** p < 0.001); daggers (†) represent main effect of stress (†† p < 0.01).

**Supplementary Table Legends**

**Supplementary Table 1:** Quantitative Real-Time PCR Primers

**Supplementary Table 2:** Inflammation-Related Gene Expression in the Fetal Brain and Placenta

Values represent fold change relative to the WT control condition (mean ± SEM). Bolded values indicate p < 0.05 with a two-way ANOVA. For the *IL10* stress x genotype interaction, expression is higher in the CCL2^-/-^ stressed condition than the CCL2^-/-^ control condition with an adjusted p = 0.0009. Placenta *IL1B*: n = 15/9, 16/8, 14/8, 13/5; *IL10*: n = 14/8, 15/7, 10/6, 10/5; *ITGAM*: 16/9, 16/8, 14/8, 13/5; *TLR2*: 14/9, 10/6, 11/7, 11/5 samples/litters in WT control, WT stress, CCL2^-/-^ control, CCL2^-/-^ stress conditions. Fetal Brain *IL1B*: n = 11/9, 13/7, 13/7, 12/5; *IL10*: n = 10/6, 12/5, 12/9, 9/5; *ITGAM*: n = 13/7, 19/8, 14/8, 13/5; *TLR2*: n = 13/8, 19/8, 15/8, 13/5 samples/litters in WT control, WT stress, CCL2^-/-^ control, CCL2^-/-^ stress conditions.
