# Supplementary figures and images for: Prenatal stress causes intrauterine inflammation and serotonergic dysfunction, and long-term behavioral deficits through microbe- and CCL2-dependent mechanisms
Source: Transl Psychiatry. 2020 Jun 16;10:191. doi: 10.1038/s41398-020-00876-5 (PMC7297973; doi:10.1038/s41398-020-00876-5)

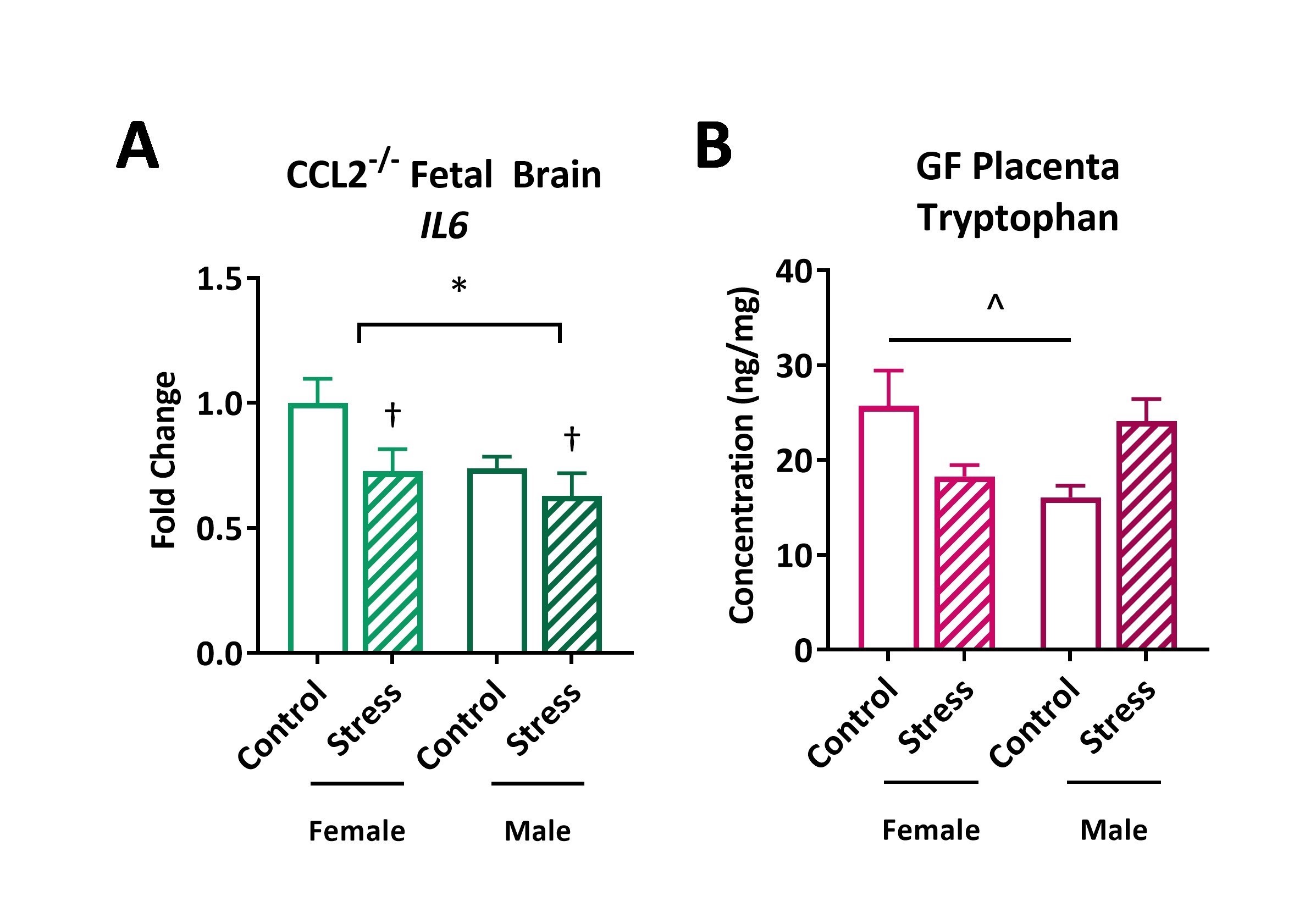

Supplement: Supplementary file 2 — Supplementary Figure 1 [file 41398_2020_876_MOESM2_ESM.jpg]

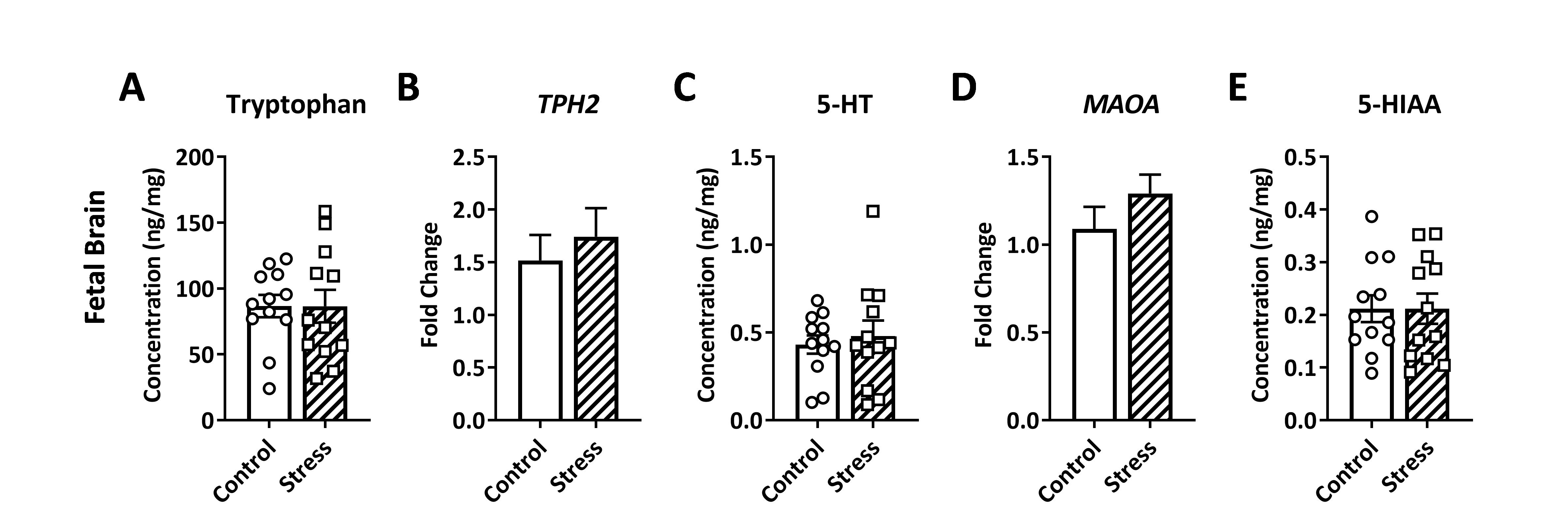

Supplement: Supplementary file 3 — Supplementary Figure 2 [file 41398_2020_876_MOESM3_ESM.jpg]

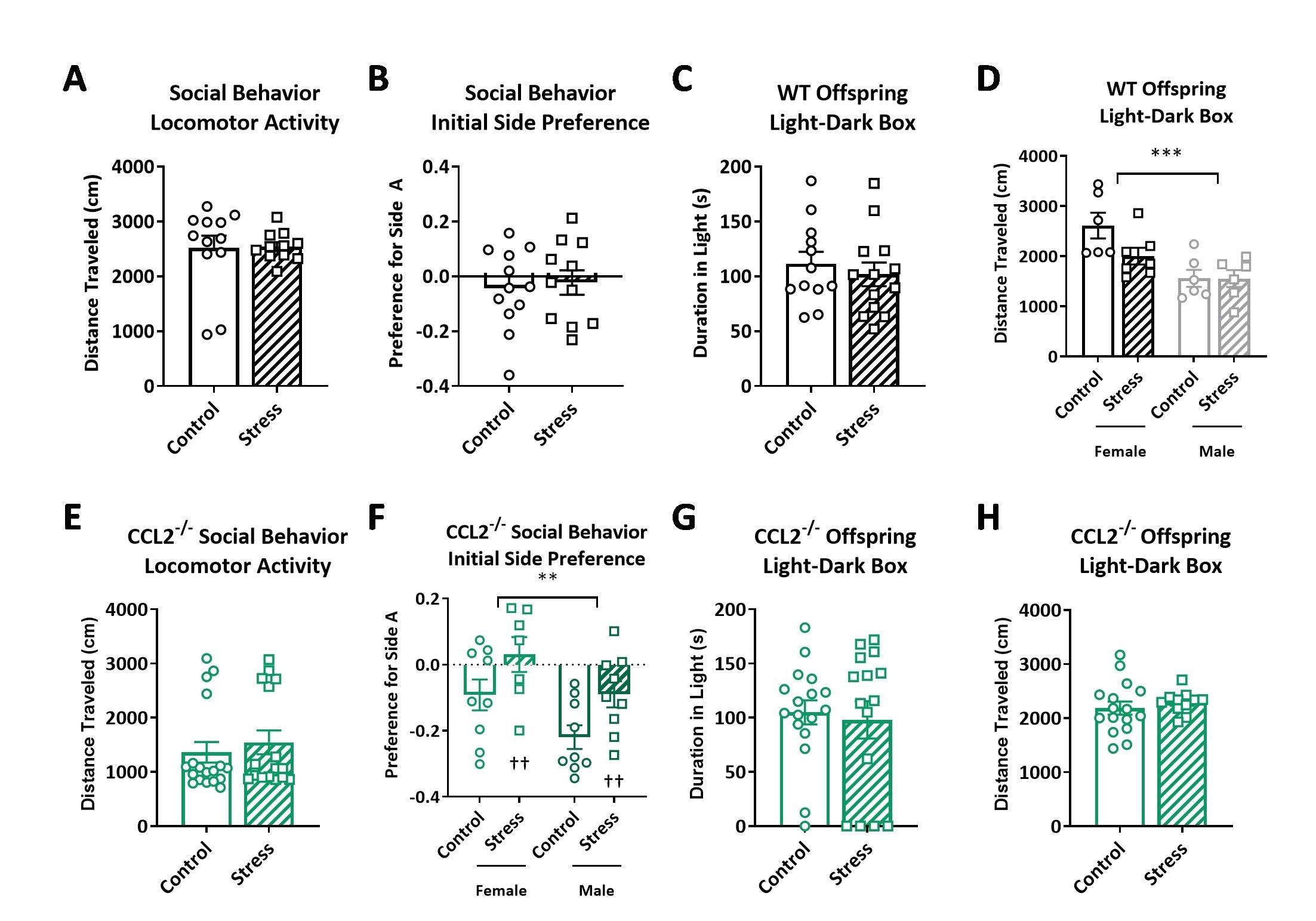

Supplement: Supplementary file 4 — Supplementary Figure 3 [file 41398_2020_876_MOESM4_ESM.jpg]
