# Supplementary material for: Prenatal stress causes intrauterine inflammation and serotonergic dysfunction, and long-term behavioral deficits through microbe- and CCL2-dependent mechanisms
Source: Transl Psychiatry. 2020 Jun 16;10:191. doi: 10.1038/s41398-020-00876-5 (PMC7297973; doi:10.1038/s41398-020-00876-5)
Supplement: Supplementary file 5 — Supplementary Tables [file 41398_2020_876_MOESM5_ESM.docx]

**Supplementary Table 1:** Quantitative Real-Time PCR Primers

|  | Gene | Assay ID |
| --- | --- | --- |
| Genes of Interest | ***CCR2*** | Mm99999051_gH |
|  | ***IL1B*** | Mm00434228_m1 |
|  | ***IL10*** | Mm01288386_m1 |
|  | ***IL6*** | Mm00446190_m1 |
|  | ***TNF*** | Mm00443258_m1 |
|  | ***TLR2*** | Mm01213946_g1 |
|  | ***TLR4*** | Mm00445273_m1 |
|  | ***TPH1*** | Mm01202614_m1 |
|  | ***TPH2*** | Mm00557715_m1 |
|  | ***MAOA*** | Mm00558004_m1 |
|  | ***IDO1*** | Mm00492590_m1 |
|  | ***SLC6A4*** | Mm00439391_m1 |
| Housekeeping Genes | ***SHDA*** | Mm01352366_m1 |
|  | ***TBP*** | Mm01277042_m1 |
|  | ***GAPDH*** | Mm99999915_g1 |

**Supplementary Table 2:** Inflammation-Related Gene Expression in the Fetal Brain and Placenta

|  | **Wild Type** | | **CCL2 Knockout** | | **Main Effect of Stress** | **Main Effect of Genotype** |  |
| --- | --- | --- | --- | --- | --- | --- | --- |
|  | **Control** | **Stress** | **Control** | **Stress** |  |  | **Interaction** |
| **Placenta** |  |  |  |  |  |  |  |
| *IL1B* | 1.00 ± 0.06 | 1.11 ± 0.07 | 1.16 ± 0.05 | 1.06 ± 0.05 | F(1,54) = 0.01  p = 0.91 | F(1,54) = 0.68  p = 0.41 | F(1,54) = 3.03  p = 0.09 |
| *IL10* | 1.00 ± 0.14 | 1.42 ± 0.24 | 0.42 ± 0.06 | 1.83 ± 0.34 | **F(1,45) = 16.78**  **p = 0.0002** | F(1,45) = 0.15  p = 0.70 | **F(1,45) = 4.97**  **p = 0.03** |
| *ITGAM* | 1.00 ± 0.09 | 0.77 ± 0.09 | 0.51 ± 0.04 | 0.55 ± 0.04 | F(1,55) = 0.38  p = 0.54 | **F(1,55) = 33.07**  **p < 0.0001** | F(1,55) = 1.26  p = 0.27 |
| *TLR2* | 1.00 ± 0.09 | 0.71 ± 0.08 | 0.80 ± 0.14 | 0.70 ± 0.04 | **F(1,42) = 3.93**  **p = 0.05** | F(1,42) = 1.29  p = 0.26 | F(1,42) = 1.01  p = 0.32 |
| **Fetal Brain** |  |  |  |  |  |  |  |
| *IL1B* | 1.00 ± 0.20 | 0.97 ± 0.08 | 1.28 ± 0.24 | 0.89 ± 0.19 | F(1,45) = 1.25  p = 0.27 | F(1,45) = 0.29  p = 0.60 | F(1,45) = 0.93  p = 0.34 |
| *IL10* | 1.00 ± 0.20 | 0.99 ± 0.20 | 0.59 ± 0.06 | 1.07 ± 0.13 | F(1,39) = 2.21  p = 0.15 | F(1,39) = 1.06  p = 0.31 | F(1,39) = 2.32  p = 0.14 |
| *ITGAM* | 1.00 ± 0.06 | 1.00 ± 0.05 | 0.93 ± 0.03 | 0.92 ± 0.04 | F(1,55) = 0.04  p = 0.84 | F(1,55) = 2.24  p = 0.14 | F(1,55) = 0.02  p = 0.89 |
| *TLR2* | 1.00 ± 0.06 | 0.98 ± 0.06 | 0.87 ± 0.05 | 0.94 ± 0.07 | F(1,56) = 0.18  p = 0.68 | F(1,56) = 2.06  p = 0.16 | F(1,56) = 0.67  p = 0.42 |

Values represent fold change relative to the WT control condition (mean ± SEM). Bolded values indicate p < 0.05 with a two-way ANOVA. For the *IL10* stress x genotype interaction, expression is higher in the CCL2^-/-^ stressed condition than the CCL2^-/-^ control condition with an adjusted p = 0.0009. Placenta *IL1B*: n = 15/9, 16/8, 14/8, 13/5; *IL10*: n = 14/8, 15/7, 10/6, 10/5; *ITGAM*: 16/9, 16/8, 14/8, 13/5; *TLR2*: 14/9, 10/6, 11/7, 11/5 samples/litters in WT control, WT stress, CCL2^-/-^ control, CCL2^-/-^ stress conditions. Fetal Brain *IL1B*: n = 11/9, 13/7, 13/7, 12/5; *IL10*: n = 10/6, 12/5, 12/9, 9/5; *ITGAM*: n = 13/7, 19/8, 14/8, 13/5; *TLR2*: n = 13/8, 19/8, 15/8, 13/5 samples/litters in WT control, WT stress, CCL2^-/-^ control, CCL2^-/-^ stress conditions.
